# Current Development Status of MEK Inhibitors

**DOI:** 10.3390/molecules22101551

**Published:** 2017-09-26

**Authors:** Ying Cheng, Hongqi Tian

**Affiliations:** Tianjin Key Laboratory of Radiation Medicine and Molecular Nuclear Medicine, Institute of Radiation Medicine, Chinese Academy of Medical Science & Peking Union Medical College, Tianjin 300192, China; chengying624@163.com

**Keywords:** MEK inhibitors, targeted therapy, combination, approved drug, clinical study, preclinical study

## Abstract

The current development status of mitogen-activated protein kinase kinase (MEK) inhibitors, including the preclinical data and clinical study progress, has been summarized in this review. Different MEK inhibitors, possessing specific physicochemical properties and bioactivity characteristics, may provide different options for patients seeking treatment for cancer. Moreover, the combination of the MEK inhibitors with other therapies—such as chemotherapy, targeted therapy, and immunotherapy—may be a promising approach for clinical use.

## 1. Introduction

The mitogen-activated protein kinase (MAPK) signaling pathway plays critical roles in the regulation of diverse cellular activities, including cell proliferation, survival, differentiation, and motility [1]. Dysregulation of the MAPK pathway occurs in more than one-third of all malignancies. The classical MAPK pathway consists of Ras (a family of related proteins which is expressed in all animal cell lineages and organs), Raf (a family of three serine/threonine-specific protein kinases that are related to retroviral oncogenes), MEK (mitogen-activated protein kinase kinase), and ERK (extracellular signal-regulated kinases), sequentially relaying proliferative signals generated at the cell surface receptors into the nucleus through cytoplasmic signaling. The MEK inhibitor targets the Ras/Raf/MEK/ERK signaling pathway, inhibiting cell proliferation and inducing apoptosis. It hence has potential in clinical use for cancer treatment, especially for those cancers induced by RAS/RAF dysfunction [2].

Owing to the widespread activation of this pathway in numerous neoplasms, MEK inhibitors have been in the process of development and study as a type of monotherapy or combination therapy with other targeted and cytotoxic drugs in a variety of clinical situations. More recently, the combination with the use of immune checkpoint inhibitors has emerged as an efficacious treatment for some cancers, expanding the efficacy of this class of agent [3].

This review summarized the recent progress of MEK inhibitors, complementary to an earlier review [4,5,6,7,8,9,10,11,12,13,14,15] but with a greater focus on those compounds that have been approved or are in clinical stages of development. We also give a brief summary of compounds in the preclinical phase.

## 2. Ras/Raf/MEK/ERK Pathway and MEK Inhibitors

Signal transduction occurs when an extracellular signaling molecule activates a specific receptor located on the cell surface. In turn, this receptor triggers a biochemical chain of events inside the cell and creates a response. The Ras/Raf/MEK/ERK pathway is one of the critical pathways involved in signal transduction, which results in the control of cell proliferation, survival, and differentiation [16,17] and plays a role in the development of multiple cancers including melanoma, non-small cell lung cancer (NSCLC), etc. MEK1 and MEK2 are closely related and participate in the Ras/Raf/MEK/ERK signal transduction cascade. Blockage of the pathway with MEK1/2 inhibitors could result in the clinical benefits for treatment of cancers with RAS/RAF dysfunction. Therefore, huge efforts have been made in past decades with respect to the development of MEK1/2 inhibitors. The first MEK inhibitor, PD098059, was reported in 1995 [18], since then a number of MEK inhibitors have been progressing into clinical stages of development. More recently, the combination of MEK with BRAF (a human gene that encodes a protein called B-Raf) inhibitors and/or other therapies has provided a new treatment option for multiple cancers.

## 3. MEK Inhibitors Approved by the US Food and Drug Administration (FDA)

Two MEK inhibitors, trametinib and cobimetinib, were approved by the FDA and the European Medicines Agency (EMA). The preclinical and clinical data for these two compounds are summarized in Table 1.

### 3.1. Trametinib

**Figure 1:**
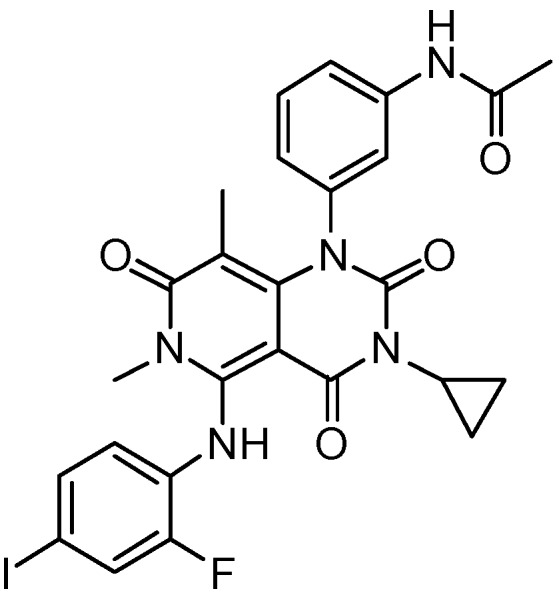
Chemical formula of trametinib.

Trametinib (GSK1120212) was the first MEK inhibitor approved by the FDA (May, 2013) for treatment of melanoma. It is an allosteric, non-ATP-competitive inhibitor with sub-nanomolar activity against purified MEK1 and MEK2 kinases (half maximal inhibitory concentration (IC_50_) of 0.7 nM [19] and 0.9 nM, respectively) and its chemical structure is shown in Figure 1.

In a preclinical study, trametinib showed high efficacy in xenograft models. For the HT-29 xenograft model, oral administration of trametinib demonstrated efficacy in inhibiting tumor growth at a dose of either 0.3 mg/kg or 1 mg/kg once daily for 14 days, and could block tumor growth almost completely at a dose of 1 mg/kg [27]. Similar findings were observed in an A549 (KRAS (proto-oncogene corresponding to the oncogene first identified in Kirsten rat sarcoma virus) mutant cell line) xenograft model with tumor growth inhibition of 92% and 87% at 5.0 mg/kg and 2.5 mg/kg respectively, with inhibition to a lesser degree with a lower dose [28].

In clinical study as a single agent, treatment with trametinib resulted in a statistically significant and clinically meaningful improvement with respect to progression-free survival (PFS) compared to standard chemotherapy. In a global multi-center, randomized, open-label and controlled trial, 322 melanoma patients with the BRAF V600E or V600K-mutant were allocated with the ratio of 2:1 into either the trametinib group or conventional chemotherapy group (dacarbazine or paclitaxel), respectively. It was reported that trametinib resulted in an improved overall response rate (22% vs. 8%) and progression-free survival (4.8 vs. 1.5 months) when compared with the group receiving cytotoxic treatment [29]. Rash, diarrhea, and peripheral edema were the main adverse events, but those were manageable. A phase I/Ib study evaluating trametinib plus docetaxel or pemetrexed in patients with advanced KRAS-mutant non-small cell lung cancer (NSCLC) showed that the primary endpoint of overall response rate (ORR) was met for both combinations [30] (ClinicalTrials.gov number, NCT01192165).

The FDA first approved the combination of trametinib and dabrafenib for patients with unresectable or metastatic melanoma with BRAF V600E or V600K mutations in January 2014, based on the results of median PFS extended from 8.8 months to 11.0 months when compared with dabrafenib monotherapy in a multi-center phase 3 trial [31,32]. The EMA approved this combination a year later for adults with unresectable or metastatic melanoma with the BRAF V600 mutation. MEK and BRAF inhibitors hence presented a new treatment method for metastatic melanoma [33].

For BRAF-mutant non-small cell lung cancer (NSCLC), the FDA gave the breakthrough designation for combination of trametinib and dabrafenib in 2015, and approved this combination in June 2017 [34] following the EMA’s approval in April [35]. The approval of this combination was based on the results from a three-cohort, multicenter, non-randomized, open-label study of patients with stage IV NSCLC. In this phase II trial, 36 treatment-naïve patients and 57 previously treated patients were assigned to the combination regimen with trametinib and dabrafenib, and 78 previously treated patients received the single agent dabrafenib. The results showed that there was an overall response rate (ORR) of more than 60%, with median PFS of longer than 9 months in the combination group, while there was an ORR of 27% with PFS of 5.5 months in the dabrafenib group [36]. The combination of MEK and BRAF inhibitors has provided a new treatment option [37].

Recently, the combination of MEK inhibitor with programmed cell death protein 1 (PD-1) inhibitors [38,39,40] has shown good prospects. Trametinib, in combination with dabrafenib and pembrolizumab, was developed by Merck Sharp & Dohme Corp. and Novartis for the treatment of melanoma in a phase 2 study. The phase 1 study has shown a manageable toxicity profile in patients with BRAF V600-mutant melanoma and the ongoing phase 2 study will further evaluate safety and efficacy of this triple combination as a first-line therapy for BRAF-mutant melanoma.

The development of triple regimens on the above approved combination with either Pembrolizumab or PDR 001 are ongoing by Merk Sharp & Dohme and Novartis, respectively, for the treatment of melanoma. A trial to assess the triple combination of trametinib, dabrafenib, and the anti-programmed death-ligand 1 (PD-L1) antibody durvalumab in 26 patients with BRAF-mutated advanced melanoma was conducted. There was an ORR of 69%, and 16 of 18 patients have ongoing responses [41].

Apart from melanoma and NSCLC, trametinib as a single agent or in combination with dabrafenib has also been tested in other types of cancers. As reported in American Society of Clinical Oncology (ASCO) recently, a two-arm phase II study with either trametinib or 5-fluororacil/capecitabine in refractory advanced biliary cancer was stopped due to the lack of efficacy in the trametinib arm. Meanwhile, a combination with dabrafenib was shown to be well tolerated in BRAF-mutated papillary thyroid carcinoma in one randomized phase II trial.

### 3.2. Cobimetinib

The second approved MEK inhibitor was cobimetinib (GDC-0973, XL518), which was developed by Exelixis and Genentech (Roche). The compound was awarded the status of orphan drug by the FDA in 2014 for malignant melanoma with the BRAFV600 mutation, and was then approved for combination treatment with vemurafenib for unresectable or metastatic melanoma with a BRAF V600E or V600K mutation in November 2015.

**Figure 2:**
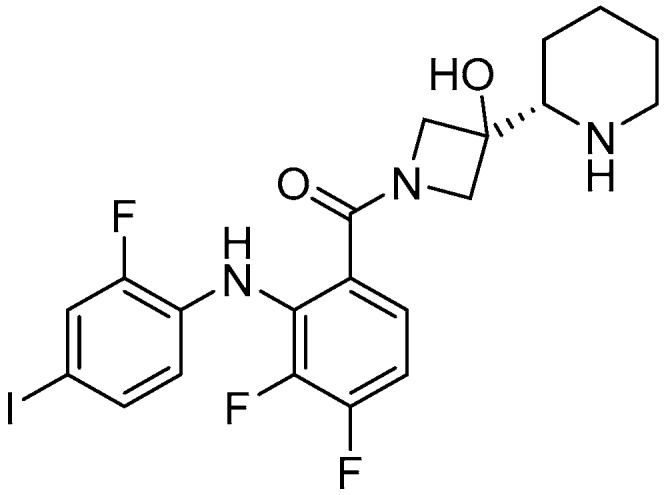
Chemical formula of cobimetinib.

The chemical structure of cobimetinib is shown in Figure 2. Cobimetinib is a potent and highly selective MEK inhibitor, with a biochemical IC_50_ of 0.9 nM against MEK1 [20]. It also showed broad efficacy in vivo in xenograft models with BRAF- and KRAS-mutated cell lines [42]. Preclinical studies demonstrated that this agent was effective in inhibiting the growth of many tumor cells bearing a BRAF mutation. In preclinical studies, cobimetinib was associated with sustained ERK/MAPK inhibition in tumor tissues, with minimal drug exposure in the brain.

In a phase 3 trial, median PFS was 12.3 months for cobimetinib plus vemurafenib versus 7.2 months for vemurafenib alone, and these data supported the use of combination as a standard first-line approach to improve survival in patients with advanced BRAF V600-mutant melanoma [43].

Currently, there are many ongoing clinical studies for cobimetinib combined with other targeted therapies. For example, there is the combination of cobimetinib with GDC-0941 (a PI3K inhibitor) or GDC-0994 (a Erk1/2 inhibitor) for metastatic solid tumors (ClinicalTrials.gov number, NCT02457793) and the combination with idasanutlin (p53-MDM2 inhibitor) and venetoclax (BCL-2 inhibitor) for the treatment of leukemia.

The combination of cobimetinib with immunotherapy is also a highly investigated topic. A phase Ib dose-escalation and dose-expansion study (ClinicalTrials.gov number, NCT01988896) achieved longer PFS, with a median of 12.0 months in the combination treatment group of cobimetinib with atezolizumab when compared with atezolizumab or cobimetinib alone in melanoma [44]. An updated phase Ib study (ClinicalTrials.gov number, NCT01656642) showed that cobimetinib + atezolizumab + vemurafenib in BRAFV600-mutant metastatic melanoma had a manageable safety profile and promising antitumor activity [45]. Cobimetinib, in combination with atezolizumab and regorafenib, was used for the treatment of colorectal cancer in the phase III trial (ClinicalTrials.gov number, NCT02788279).

The clinical study results of clinical activity and safety of cobimetinib (cobi) and atezolizumab (atezo) in colorectal cancer (CRC) reported that the combination of cobi and atezo in CRC is well tolerated at the maximum administered doses. These results show that patients with MSS CRC can respond to the combination of cobi and atezo, and provide support for continued evaluation of the combination [46] (ClinicalTrials.gov number, NCT01988896).

## 4. MEK Inhibitors under Clinical Development

**Figure 3:**
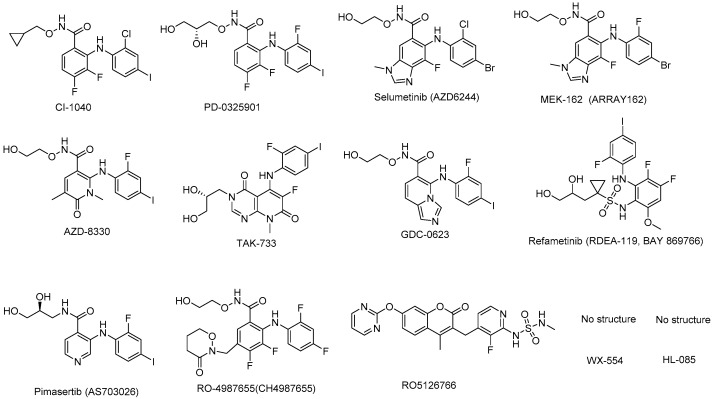
MEK inhibitors in clinical study.

The MEK inhibitors in clinical trials—including their efficacy, therapeutic indications, sponsors, and status—are shown in Table 2. At the same time, the chemical structures of the compounds are presented in Figure 3.

### 4.1. CI-1040 (PD184352)

CI-1040 was the first MEK inhibitor run in the clinical stage by Pfizer/Warner-Lambert [47]. This compound presented a novel series of benzhydroxamate esters derived from their precursor anthranilic acids as MEK inhibitors. CI-1040 was developed in phase II for breast cancer, colorectal cancer, lung cancer, and pancreatic cancer, but it was found that there was poor exposure related to its poor solubility and rapid clearance [57], leading to insufficient antitumor activity in two phase II studies completed in 2003 (ClinicalTrials.gov number, NCT00034827, NCT00033384). Hence, to resolve the problems of this compound, the second compound PD-0325901 had been developed into clinical trial.

### 4.2. PD-0325901

PD-0325901 is a derivative of the MEK inhibitor CI-1040. The optimization of the hydroxamate side chain of CI-1040 for the improvement of solubility and exposure with oral doses resulted in the discovery of the clinical candidate PD-0325901 by Pfizer/Warner-Lambert. The administration of oral dose (PO) or intravenous injection (IV) of PD-0325901 induced in a dose-dependent decrease in phosphorylation of MAPK (pMAPK) in liver and lung caused by MEK inhibition. Inhibition of pMAPK in liver was generally comparable between the routes of administration, whereas inhibition of pMAPK in lung occurred for a longer duration following IV administration of PD-0325901, possibly due to higher PD-0325901 plasma maximum concentration (C_max_) [58,59].

PD-0325901 failed in its phase II clinical trials for the treatment of KRAS mutant non-small cell lung cancer by not meeting its primary efficacy end-point (ClinicalTrials.gov number, NCT00174369) [60]. A phase I/II study for the treatment of melanoma, colonic neoplasms and breast neoplasms had been terminated in 2007 due to musculoskeletal, neurological, and ocular toxicity (ClinicalTrials.gov number, NCT 00147550) [61]. Furthermore, the combination of PD-325901 with palbociclib for the same indication is still ongoing to date (ClinicalTrials.gov number, NCT 02022982). A phase 2 study in adolescents and adults with neurofibromatosis type-1 is ongoing and is expected to be completed at the end of 2018 (ClinicalTrials.gov number, NCT 02196471). There are another two phase I or I/II studies ongoing in colorectal cancer or KRAS mutated malignancies (ClinicalTrials.gov number, NCT02510001, NCT02039336); both are combinations with target agents or chemotherapy.

### 4.3. Selumetinib (ARRY-142886; AZD6244)

Selumetinib (AZD6244) is an effective, high selective, non-ATP-competitive MEK1 inhibitor which was developed by Array BioPharma and then licensed to AstraZeneca.

In 2004, Array BioPharma and AstraZeneca co-developed selumetinib for clinical study with several phase I and II clinical trials carried out for solid tumors as monotherapy [59,62,63,64,65]. Unfortunately, it was discontinued in phase II trials due to its failure to distinguish from temozolomide.

Randomized phase II studies that compared selumetinib with conventional cytotoxic chemotherapy in a wide array of malignancies have also been performed. Selumetinib showed no superiority when compared with temozolomide in patients with chemotherapy-naive melanoma, when compared with pemetrexed in patients with NSCLC who had not responded to first-line and second-line therapies, and when compared with capecitabine in patients with pancreatic cancer and colorectal cancer. Nevertheless, an antitumor activity of selumetinib was detected in each of these comparative studies.

Several clinical trials on selumetinib in combination with other cancer drugs have been conducted. A phase Ib study of combination of selumetinib and cyclosporine A (CsA) in patients with advanced solid tumors with an expansion cohort in metastatic colorectal cancer (mCRC) showed that the combination therapy appeared to be well tolerated, with evidence of activity in mCRC (ClinicalTrials.gov number, NCT02188264) [66]. Selumetinib in combination with sorafenib in advanced hepatocellular carcinoma (HCC) is also under study (ClinicalTrials.gov number, NCT01029418) [67]. A randomized Phase II study for KRAS-mutated NSCLC did show very promising results for selumetinib + docetaxel vs. docetaxel, with significant improvement in median PFS (5.3 vs. 2.1 months, *p* = 0.014) and objective response rate (37% vs. 0%, *p* < 0.0001) when compared with the docetaxel monotherapy cohort, and the overall survival benefit is 9.4 vs. 5.2 months (*p* = 0.21), with higher toxicity and the overall survival benefit (9.4 vs. 5.2 months, *p* = 0.21), with high toxicity. However, such positive efficacy could not repeat in the multinational phase III clinical trial. A total of 510 KRAS-mutated NSCLC patients were randomized 1:1 to receive selumetinib + docetaxel or placebo + docetaxel. The result showed that addition of selumetinib to docetaxel did not improve progression-free survival compared with docetaxel alone (ClinicalTrials.gov number, NCT01933932) [68]. Combination therapy with selumetinib plus dacarbazine has also been compared with placebo plus dacarbazine as first-line treatment in patients with BRAF mutated melanoma in a phase II study. While markedly improved PFS was observed with the addition of selumetinib to the therapeutic regimen, no overall survival benefit was demonstrated.

Recently, a phase I trial in pediatric patients with recurrent or refractory low-grade glioma has been reported. In this study, 25 subjects received a median of 13 cycles (range: 1–26). Fourteen (37%) completed all protocol treatment (26 cycles (*n* = 13), 13 cycles (*n* = 1)) with at least stable disease; 2-year progression-free survival at the RP2D was 69 ± SE (standard error) 9.8% [69]. The study showed that selumetinib had promising antitumor activity in children with low-grade glioma. In the early phase 2 result, selumetinib was effective in treating children with recurrent/refractory low-grade glioma (LGG), including those with neurofibromatosis type 1 (NF-1)-associated LGG and pilocytic astrocytomas (PA) harboring BRAF V600E mutation or BRAF-KIAA 1549 fusion. Larger prospective studies are necessary to determine the specific role of this agent in treating children with LGG harboring specific molecular aberrations in the future [70].

### 4.4. Binimetinib (MEK162, ARRY-438162)

Binimetinib (MEK162, ARRY-438162) is an orally bioavailable, highly selective, non-ATP-competitive MEK inhibitor that has the potential to treat a range of malignant diseases. Binimetinib, 5-((4-bromo-2-fluorophenyl)amino)-4-fluoro-*N*-(2-hydroxyethoxy)-1-methyl-1*H*-benzo[*d*]imidazole-6-carboxamide, was discovered by Array BioPharm., and was then co-developed by Array BioPharm/Novartis. In preclinical studies, binimetinib either alone or in combination with other agents showed significant antitumor activities in cell lines and animal models [71].

Binimetinib was investigated in phase III trials as a single agent or in combination with other anticancer agents. Common adverse events (AEs) included rash, nausea, vomiting, diarrhea, peripheral edema, and fatigue [72].

The open-label and non-randomized phase II study assessed the safety and efficacy of single-agent binimetinib in adult patients with locally advanced and unresectable or metastatic malignant cutaneous melanoma, harboring BRAF V600E or NRAS (a member of the RAS gene family) mutations (ClinicalTrials.gov number, NCT01320085). The resulting data supported clinical activity of binimetinib in patients with NRAS-mutated and BRAF-mutated metastatic melanoma. Binimetinib was the first targeted therapy to show activity in patients with NRAS-mutated melanoma and offered a potential option for kinds of cancer with few effective treatments [71,73].

Then, a randomized phase III, open label, multicenter, two-arm study was designed to compare the efficacy of binimetinib versus dacarbazine in patients with advanced unresectable or metastatic NRAS mutation-positive melanoma (NCT01763164). The data showed that the ORR was 15% vs. 7% and median PFS was 2.8 months (95% CI 2.8–3.6) vs. 1.5 months (1.5–1.7) in the binimetinib group and dacarbazine group, respectively (hazard ratio 0.62 (95% CI (confidence interval) 0.47–0.80); one-sided *p* < 0.001) [74]. PFS by binimetinib was statistically improved but not clinically significant (2.8 vs. 1.5 months). A new drug application (NDA) application for binimetinib as monotherapy for NRAS-mutated melanoma was withdrawn finally from the FDA. Another prospective, randomized, open label, multi-center, parallel group, three-arm phase III study is in progress to study the efficacy and safety of binimetinib with LGX818 (encorafenib) compared vemurafenib and LGX818 monotherapy in locally advanced unresectable or metastatic melanoma with BRAF V600 mutation (ClinicalTrials.gov number, NCT01909453).

Other plans for binimetinib combinations with immunotherapy (e.g., with pembrolizumab and encorafenib for the treatment of malignant melanoma, with nivolumab, LGX818, and ipilimumab for the treatment of metastatic melanoma, etc.) are also underway.

### 4.5. AZD-8330 (ARRY-424704)

AZD8330 is an orally active, selective MEK inhibitor with potential antineoplastic activity. The structure core for this compound was 6-oxo-1,6-dihydropyridazine, which presents a different class of MEK inhibitors [75]. AZD-8330 was being developed for non-ATP-competitive MEK1/2 inhibitors, with an IC_50_ of 7 nM [76].

AZD8330 was employed as a single agent, and the results from phase I clinical trial for the treatment of solid tumors (ClinicalTrials.gov number, NCT00454090) showed that common reported toxicities included acneiform dermatitis, fatigue, diarrhea, and vomiting. Four patients experienced dose-limiting toxicities: mental status changes (40 mg once daily; 2/9 patients and 60 mg once daily; 1/3) and rash (20 mg BID (twice daily); 1/9). The maximum tolerated dose was defined as 20 mg BID. The exposure of AZD8330 increased approximately proportionally with dosage across a dose range of 0.5–60 mg once-daily. Phosphorylated ERK levels in peripheral blood mononuclear cells were measured and target inhibition was confirmed. AZD8330 demonstrated a manageable toxicity profile with fewer class-effect AEs compared with other MEK inhibitors [77]. No more clinical studies have been reported recently.

### 4.6. TAK-733

TAK-733 is an orally bioavailable, non-ATP-competitive small-molecule inhibitor of MEK1/2 with potential antineoplastic activity. TAK-733 is highly potent and selective MEK allosteric site inhibitor with IC_50_ of 3.2 nM, and potent enzymatic and cell activity with an EC_50_ (concentration for 50% of maximal effect) of 1.9 nM against ERK phosphorylation in cells [51]. TAK-733 reveals broad antitumor activity in mouse xenograft models of human cancer including models of melanoma, colorectal, NSCLC, and pancreatic and breast cancer.

Phase I clinical study (ClinicalTrials.gov number, NCT00948467) of TAK-733 has been developed by Millennium Pharmaceuticals Inc., and the results from the dose-escalation phase I showed that the maximum tolerated dose was 16 mg. Common drug-related AEs included dermatitis acneiform, diarrhea, and increased blood creatine phosphokinase. TAK-733 demonstrated a generally manageable toxicity profile and limited antitumor activity [78]. No further investigations have been published recently.

### 4.7. GDC-0623

GDC-0623, with the chemical name of (1-(5-((2-fluoro-4-iodophenyl)amino)imidazo[1,5-*a*]pyridin-6-yl)-2-(2-hydroxyethoxy)ethan-1-one), is a potent, orally active, selective non-ATP-competitive MEK inhibitor (MEK1, Ki = 0.13 nM, + ATP). It was developed by Genentech, and has a novel structure of imidazopyridine [52]. GDC-0623 has broad potency in cell-based assays, particularly in both KRAS and BRAF mutant cancer cell lines, with corresponding efficacy in xenograft tumor models. It exhibited higher activity in KRAS mutant tumors than BRAF mutant tumors [79]. In preclinical pharmacokinetics and efficacy assessments, GDC-0623 showed low clearance and a low volume of distribution. The results of safety, tolerability, and pharmacokinetics from its phase I study were revealed in 2014 (ClinicalTrials.gov number, NCT01106599).

### 4.8. Refametinib (RDEA-119, BAY-869766)

Refametinib, discovered by Ardea Biosciences and developed by Bayer, is a potent and orally bioavailable, non-ATP-competitive inhibitor with a low ability to accumulate in brain and other neural tissues. The IC_50_ of refametinib against MEK1 was 19 nM [53]. It was selected for clinical development due to its potency and favorable pharmacokinetic profile.

Several phase I or I/II or phase II clinical trials had been conducted for single or combination therapy. One phase I/II study concluded that refametinib plus gemcitabine was well tolerated, with a promising objective response rate [80]. One phase II clinical study of the combination therapy with rafametinib and sorafenib for the treatment of RAS-mutated HCC has been carried out as the first line systemic treatment due to its high prevalence of constitutive activation of MAPK pathway in HCC. The results showed the efficacy benefit; among 70 enrolled patients, three had confirmed partial response and 25 had prolonged stable disease [81]. However, this combination was poorly tolerated, with several severe adverse events reported and almost all patients required dose modifications due to drug toxicity.

### 4.9. Pimasertib (AS703026)

Pimasertib, developed by Merck KGaA and known as AS703026 or MSC1936369B, is a selective, orally bioavailable, non-ATP-competitive MEK1/2 inhibitor with potent antitumor activity in cell lines and xenograft models with constitutive activation of the MAPK pathway. Its structure, including (2-fluoro-4-iodophenyl) amino, core structure pyridine, and side chain (*S*)-*N*-(2,3-dihydroxypropyl)acetamide, is different from other MEK inhibitors. The first-in-human trial reported pharmacokinetics (PK) and pharmacodynamics (PD) of pimasertib in patients with advanced solid tumors. Pimasertib exhibited a favorable PK profile in patients with solid tumors, and target engagement demonstrated by phospho-ERK (pERK) inhibition in peripheral blood mononuclear cell (PBMC) was observed [82,83]. Pimasertib showed clinical activity in dose-dependent manner associated with target inhibition. Sustained responses were observed mostly in BRAF or NRAS-mutated melanoma [84,85]. Currently, a few phase I/II studies are ongoing to evaluate pimasertib in the setting of advanced or metastatic solid tumors including ovarian cancer, NRAS-mutated cutaneous melanoma, ovarian cancer, breast cancer, NSCLC, hepatocellular carcinoma, metastatic colorectal cancer, and pancreatic adenocarcinoma.

### 4.10. RO4987655 (CH4987655)

RO4987655, possessing a unique 3-oxo-oxazinane ring structure at the 5-position of the benzamide core structure [54], was developed by Hoffman–La Roche. RO4987655 is an orally active small molecule, targeting MEK1 with potential antineoplastic activity. It was designed based on the X-ray crystal structure information of the target enzyme and then given multidimensional optimization including metabolic stability, physicochemical properties, and safety profiles.

RO4987655 exhibited slow dissociation from the MEK1 enzyme, remarkable antitumor efficacy both as monotherapy and combination therapy in vivo, desirable metabolic stability, and insufficient MEK inhibition in mouse brain, implying few central nervous system (CNS)-related side effects in humans.

An excellent PK profile and clear target inhibition in PBMC were demonstrated in one phase I study with healthy volunteers [86], and also showed manageable toxicity, a favorable PK/PD profile, and promising preliminary antitumor activity with heavily pretreated patients [76,87,88], which has been further investigated in specific populations of patients with RAS and/or RAF mutation driven tumors (ClinicalTrials.gov number, NCT00817518).

### 4.11. RO5126766

RO5126766, known as CH5126766, is a dual Raf/MEK inhibitor specifically for the kinase activities of Raf and MEK, resulting in the blockage of target gene transcription that promotes malignant transformation of cells. RO5126766 specifically inhibits the kinase activities of Raf and MEK, resulting in the inhibition of target gene transcription that promotes malignant transformation of cells. RO5126766, bearing a sulfamide moiety instead of aniline in coumarin, was identified as a clinical compound with enhanced inhibitory activity, satisfactory PK/PD profiles, and manageable toxicity [89]. More studies are needed to further clarify the safety and efficacy of this agent, as well as this novel class of MEK-RAF inhibitors in various cancers.

### 4.12. WX-554

WX-554 is a selective, noncompetitive MEK1/2 inhibitor entering preliminary human studies. Phase I pharmacokinetic and pharmacodynamic study showed that WX-554 was well tolerated, with the recommended phase 2 dose being 75 mg twice weekly [55]. Unfortunately, two dose-escalation phase I/II studies in patients with advanced solid tumors were terminated as reported (ClinicalTrials.gov number, NCT01859351, NCT01581060).

### 4.13. HL-085

HL-085 is an orally active, selective MEK inhibitor with significant inhibitory activity against MEK kinase and an IC_50_ value of 1.9–10 nM. In vitro, the IC_50_ values of tumor cell lines were 0.41–6.2 nM in A375, 0.1–7.8 nM in Colo205 and 0.88–2.9 nM in HT29, respectively. Oral dosing of HL-085 (1 mg/kg, QD, 21 days) in BRAF-mutant Colo 205 and A375 xenograft models showed a high tumor growth inhibition (TGI) value (70–76%, 60–70%). HL-085 is effective in inhibiting tumor proliferation in other tumor cells as well. Currently, HL-085 is under phase I clinical study.

## 5. MEK Inhibitors in Preclinical Development

**Figure 4:**
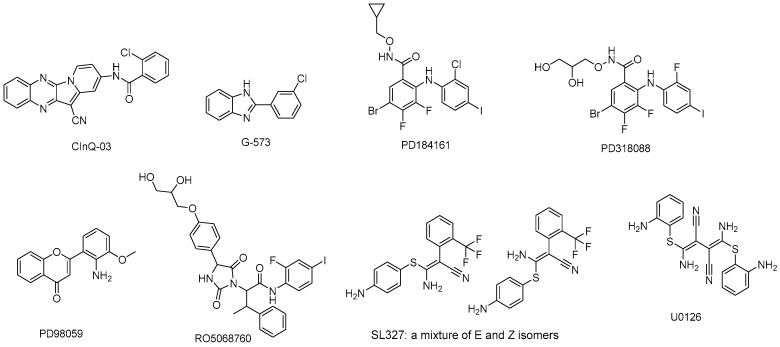
MEK inhibitors in preclinical study.

There are several MEK inhibitors in preclinical development (Table 3) and the structures are shown in Figure 4.

### 5.1. CInQ-03

CInQ-03 is a novel and specific MEK inhibitor both in vitro and in vivo. CInQ-03 has a distinct chemical structure compared with current MEK inhibitors, such as PD318088, PD184352, PD0325901, and selumetinib. The compound has similar binding affinity and displays almost the same binding mode with known inhibitors. CInQ-03 binds deeply into the binding pocket in a manner similar to the crystal ligand; the docking and experimental data suggest that CInQ-03 shows an ability to inhibit MEK1/2.

A xenograft mouse model indicated that administration of CInQ-03 at 1 or 5 mg/kg for 11 days could suppress colon cancer cell growth significantly without toxicity. Furthermore, CInQ-03 could decrease phosphorylation level of ERKs in a cell-based assay, which was highly consistent with the results in vivo. Based on these findings, CInQ-03 is considered and is being developed as a novel MEK inhibitor [90].

### 5.2. G-573

G-573 is a potent and selective allosteric inhibitor of MEK. Structural and functional analysis illustrates that G-573 could form a strong hydrogen bond with MEK kinase. The IC_50_ value for pERK inhibition in HCT116 tumor by G-573 was estimated to be 0.406 μM, and ED_50_ values in HCT116 and H2122 mouse xenograft models were estimated to be 4.6 and 1.9 mg/kg/day, respectively [91].

### 5.3. PD184161

PD184161 is an orally-active MEK inhibitor which could inhibit MEK activity more effectively (IC_50_ = 10–100 nM) in a time- and concentration-dependent manner than PD098059 or U0126. PD184161, a structurally related analog of CI-1040 but distinct from PD098059 and U0126, joins the mechanistic class of agents that inhibit the downstream phosphorylation of ERK through their direct effects on MEK. Unlike PD098059 and U0126, PD184161 has the obvious advantages of solubility and oral bioavailability. It exerts antitumor effects against HCC (hepatic cellular cancer) in vitro and in vivo which appears to correlate with suppression of MEK activity. PD184161 is unable to suppress MEK activity in HCC xenografts in the long term [92,93].

### 5.4. PD318088

PD318088, an analog of CI-1040 with a biarylamine structure, is a novel non-ATP-competitive MEK1 inhibitor. The compound-binding site of PD318088 is adjacent to the ATP binding site. Its potency in inhibiting MEK1 activation is not affected by ATP concentration, suggesting that PD318088 is not competitive with ATP [94].

### 5.5. PD98059

PD98059 is a potent and selective non-ATP-competitive MEK1 inhibitor. It mediates its inhibitory properties by binding to the ERK-specific MAP kinase MEK, therefore preventing phosphorylation of ERK1/2 (p44/p42 MAPK) by MEK1/2. PD98059 can interact with GST-MEK1 or partially activate MEK, but does not inhibit the MAPK homologues JNK and P38 (D.R. Alessi and P. Cohen, personal communication) [95]. In addition, PD98059 can highly inhibit MEK in a selective manner, but not the other kinases such as Raf kinase, cAMP (cyclic Adenosine monophosphate)-dependent kinase, protein kinase C, v-Src (a gene found in Rous sarcoma virus), epidermal growth factor (EGF) receptor kinase, and phosphatidyl inositol kinase 3 [96,97].

### 5.6. RO5068760

RO5068760, a substituted hydantoin, is a potent, highly selective, non-ATP-competitive MEK1/2 inhibitor. In vitro, RO5068760 could inhibit MEK1 kinase activity potently with an IC_50_ value of 0.025 ± 0.012 μmol/L in Raf/MEK/ERK cascade assay. It also shows significant efficacy in a broad spectrum of tumors via the activation of aberrant mitogen-activated protein kinase pathway. RO5068760 shows superior efficacy in tumors harboring the BRAF V600E mutation [98]. The assessment study of target suppression in healthy volunteers showed a peak time (t_max_) of two hours and biphasic disposition with reaction half-time (T_1/2_) of 5~9 h. The inhibition of pERK was relatively modest, with mean maximal pERK suppression of 55% [99].

### 5.7. U0126

U0126 is a potent and selective non-competitive MEK inhibitor, inhibiting MEK1 and MEK2 (IC_50_ values of 70 nM and 60 nM, respectively). It could inhibit ERK phosphorylation up to 80% in astrocytes. Surprisingly, U0126 also causes profound depletion of ATP in glucose-deprived cells, leading to death by necrosis [100]. It was reported and demonstrated [101] that U0126 could inhibit the cellular target MEK, and have an antiviral potential not only in cell culture in vitro, but also in the mouse mode in vivo. A promising approach might need to be considered and evaluated for MEK inhibitors as new antivirals against influenza.

### 5.8. SL327

SL327 is a homolog of U0126, with a mixture of E and Z isomers. SL327 inhibits MEK1 and MEK2, with IC_50_ values of 0.18 μM and 0.22 μM, respectively. In vivo, the study showed that the combination of SL327 with sunitinib malate induced significant additive suppression of doxorubicin-resistant anaplastic thyroid carcinoma (ATC) tumor growth [102].

## 6. Conclusions

In recent years, MEK inhibitors have been discovered and developed very rapidly. Trametinib became the first approved drug among MEK inhibitors, and cobimetinib soon followed suit. MEK inhibitors as single agents or in combination with other therapies have shown to be efficacious in treating melanoma, lung cancer, and colorectal cancer.

According to available clinical data, MEK inhibitor plus a BRAF inhibitor could be a superior therapy. However, a proportion of patients being treated with the combination may not achieve optimal results.

Some preclinical studies show that a MEK inhibitor plus a BRAF inhibitor or other targeted agents may modulate the immunization and enhance immune activation. To further improve the efficacy, the combination of an MEK inhibitor plus a BRAF inhibitor or other targeted agents and immunotherapy could represent a promising form of cancer treatment.

However, there are still concerns regarding toxicity, which is one of the obstacles for the development of MEK inhibitors. MEK inhibitor treatment is still considered one of the most promising areas in cancer research. Novel small molecular inhibitors are expected to become the new breakthrough in cancer treatment. Moreover, dual inhibition of MEK and RAF kinase offers advantage in terms of both increased efficacy and minimized toxicity, and may be a prospective therapeutic strategy in targeting the MARK pathway in the near future.

## Figures and Tables

**Table 1:** Preclinical and clinical data for trametinib and cobimetinib.

Item		Trametinib	Cobimetinib
Structure		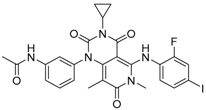	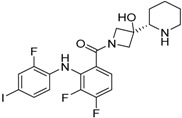
BCS ^1^ class		BCS II (high permeability, low solubility)	BCS I (high permeability, high solubility)
Salt form		dimethyl sulfoxide solvate (1:1)	hemifumarate
Molecule weight (free base)		615.4	531.32
In vitro (enzyme) [19,20]	MEK ^2^ kinase	MEK1 IC_50_^3^ = 0.7 nM;	MEK1 IC_50_ = 0.95 nM;
		MEK2 IC_50_ = 0.9 nM	MEK2 IC_50_ = 199 nM
In vitro (cell potency) [21,22]	A375	0.74 nM	5 nM
In vivo efficacy (xenograft) [19,20]	A375	TGI ^4^ = 60% @ 0.1 mpk; 14 days;	TGI = 87% @ 3 mg/kg, 21 days, QD ^5^;
		TGI = 102% @ 0.3 mpk; 14 days; TGI = 118% @ 3 mpk; 14 days	TGI = 106% @ 5 mg/kg, 21 days, QD
Pharmacokinetics (rats) [23,24]	C_max_^6^	2.7 μM (3 mg/kg, mice);	0.997 μM (30 mg/kg, male rats)
		0.47 μM (3 mg/kg, rats)	
	T_max_^7^	4 h (mice, 3 mg/kg, 14 days, repeat);	2 h (30 mg/kg, male rats)
		4 h (rats, 1 mg/kg, 21days, repeat)	
	T_1/2_^8^	3.8 h (mice);	5.56 h (30 mg/kg, male rats)
		5.5 h (rats)	
	protein binding rate	97.40% (human)	98.8% (5 μM, dog);
			93.5% (5 μM, human)
Toxicokinetics (rats) [21,22]	T_max_	4 h (male rats, 0.1667 mg/kg, week 4)	N/A
	C_max_	10.1 ng/mL (male rats, 0.1667 mg/kg, week 4)	39.9 ng/mL (male rats, 3 mg/kg, day 22)
	AUC_0-t_^9^	188 ng·h/mL (male rats, 0.1667 mg/kg, week 4)	244 ng·h/mL (male rats, 3 mg/kg, day 22)
Clinical PK [25,26]	MTD ^10^	3 mg/day (QD)	60 mg/day, QD, 21/7;
			100 mg/day, QD, 14/14
	T_max_	1.5 h (2 mg, QD)	2.4 h (60 mg, QD)
	C_max_	22.2 ng/mL (2 mg, QD)	273 ng/mL (60 mg, QD)
	T_1/2_	4–5 d (2 mg, QD)	43.6 h (60 mg, QD)
	CL/F ^11^	5.4 L/h (2 mg, QD)	13.8 L/h (60 mg, QD)
	AUC	370 ng·h/mL (0-t, day 15, 2 mg, QD)	4340 ng·h/mL (60 mg, QD)
	period/cycle	21 days/7 days	21 days/7 days
	absolute bioavailability	72% (2 mg, QD)	46% (20 mg, QD)
	recommended dose	2 mg, QD	60 mg, QD
Adverse reactions [25,26]		rash, diarrhea, fatigue, peripheral edema, nausea, and dermatitis acneiform	gastrointestinal disorders, rash, pyrexia, increased blood CPK ^12^, chorioretinopathy

^1^ BSC: biopharmaceutics classification system. ^2^ MEK: mitogen-activated protein kinase kinase. ^3^ IC_50_: half maximal inhibitory concentration. ^4^ TGI: tumor growth inhibition. ^5^ QD: once a day. ^6^ C_max_: peak concentration. ^7^ T_max_: peak time. ^8^ T_1/2_: elimination half-life. ^9^ AUC_0-t_: Area under the plasma drug concentration-time curve. ^10^ MTD: maximum tolerated dose. ^11^ CL/F: apparent plasma clearance. ^12^ CPK: creatine phosphokinase.

**Table 2:** MEK inhibitors in clinical trials.

MEK Inhibitor	Target	IC_50_	Indications	Company	Clinical Phase
CI-1040 (PD184352) [47]	MEK1/2	2.3 nM	breast cancer, colorectal cancer, lung cancer, and pancreatic cancer	Pfizer	Phase II
PD0325901 [47]	MEK1/2	0.33 nM	melanoma, colonic neoplasms, breast neoplasms, carcinoma, NSCLC ^1^	Pfizer	Phase II
Selumetinib (AZD6244) [48]	MEK1	14 nM	melanoma, NSCLC	Array BioPharma and AstraZeneca	Phase III
MEK162 [49]	MEK1/2	12 nM	BRAF ^2^ or NRAS ^3^ mutant melanoma	Array Biopharma/Novartis	Phase III
AZD8330 [50]	MEK1/2	7 nM	advanced solid tumors	AstraZeneca	Phase I
TAK-733 [51]	MEK1/2	3.2 nM	advanced non-hematologic malignancies, advanced metastatic melanoma	Millennium Pharmaceutical, Inc./Takeda Pharmaceutical Company Limited	Phase I
GDC-0623 [52]	MEK1/2	0.13 nM	metastatic solid tumors	Genentech	Phase I
Refametinib (RDEA119; BAY 869766) [53]	MEK1/2	19 nM/47 nM	hepatocellular cancer, melanoma, colorectal cancer	Ardea Biosciences/Bayer	Phase II
Pimasertib (AS703026)	MEK1/2	5–11 nM	colorectal cancer, multiple myeloma	Merck and Co.	Phase II
RO4987655 (CH4987655) [54]	MEK1	42 nM	neoplasms	Hoffman-La Roche	Phase I
RO5126766 [54]	RAF/MEK1/2	160 nM	neoplasms	Hoffmann-La Roche	Phase I
WX-554 [55]	MEK1/2	4.7 nM/10.7 nM	advanced solid tumors	Wilex, AG. Germany	Phase I/II (terminated)
HL-085 [56]	MEK1	1.9–10 nM	no data	Binjiang Pharma	Phase I

^1^ NSCLC: non-small-cell lung cancer. ^2^ BRAF: a human gene that encodes a protein called B-Raf. ^3^ NRAS: a member of the RAS gene family.

**Table 3:** MEK inhibitors in preclinical development

MEK Inhibitor	Target	IC_50_	Current Sponsor	Research Progress
CInQ-03	MEK1/2	5/10 μM	No data	in preclinical study
G-573	MEK	No data	Genentech	in preclinical study
PD184161	MEK	10–100 nM	Pfizer	in preclinical study
PD318088	MEK1	No data	Pfizer Global Research & Development	in preclinical study
PD98059	MEK1	2 μM	No data	in preclinical study
RO5068760	MEK1	0.025 ± 0.012 μM	Hoffmann-La Roche, Inc.	in preclinical study
U0126	MEK1/2	0.07 μM/0.06 μM	No data	in preclinical study
SL327	MEK1/2	0.18 μM/0.22 μM	No data	in preclinical study
